# A chromosomal reference genome sequence for the malaria mosquito, *Anopheles maculipalpis*, Giles, 1902

**DOI:** 10.12688/wellcomeopenres.22988.1

**Published:** 2024-09-26

**Authors:** Nil Rahola, Diego Ayala, Lemonde B. A. Bouafou, Boris K. Makanga, Harriet F. Johnson, Haynes Heaton, Martin G. Wagah, Joanna C. Collins, Ksenia Krasheninnikova, Sarah E. Pelan, Damon-Lee B. Pointon, Ying Sims, James W. Torrance, Alan Tracey, Marcela Uliano-Silva, Jonathan M. D. Wood, Katharina von Wyschetzki, Wellcome Sanger Institute Scientific Operations: Sequencing Operations, Shane A. McCarthy, Daniel E. Neafsey, Alex Makunin, Mara K. N. Lawniczak

**Affiliations:** 1 MIVEGEC, Univ. Montpellier, CNRS, IRD, Montpeillier, France; 2 ESV, Centre Interdisciplinaire de Recherches Médicales de Franceville (CIRMF), Franceville, Gabon; 3 Medical Entomology Unit, Institut Pasteur de Madagascar, Antananarivo, Antananarivo Province, Madagascar; 4 Département de Biologie et Écologie Animale, Institut de Recherche en Écologie Tropicale, Libreville, Gabon; 5 Scientific Operations, Wellcome Sanger Institute, Hinxton, England, UK; 6 CSSE, Auburn University, Auburn, Alabama, USA; 7 Tree of Life, Wellcome Sanger Institute, Hinxton, England, UK

**Keywords:** Anopheles maculipalpis, African malaria mosquito, genome sequence, chromosomal

## Abstract

We present a genome assembly from an individual female *Anopheles maculipalpis* (the malaria mosquito; Arthropoda; Insecta; Diptera; Culicidae). The genome sequence is 224 megabases in span. Most of the assembly is scaffolded into three chromosomal pseudomolecules with the X sex chromosome assembled. The complete mitochondrial genome was also assembled and is 15.4 kilobases in length.

## Species taxonomy

Animalia; Arthropoda; Insecta; Diptera; Culicidae; Anophelinae; Anopheles; *Anopheles maculipalpis*; Giles, 1902 (NCBI txid:1496333).

## Background

The mosquito *Anopheles maculipalpis* (Giles, 1902) has a very large distribution throughout Africa and is also present in the islands of South-West Indian Ocean ^1– 4^. It is a savannah species found up to 1000 metres above sea level in Central Africa ^1, 5^. Its larvae develop in poorly oxygenated environments, typically among abundant aquatic vegetation. Their habitats are sunny with shallow, stagnant water, often muddy. They vary greatly in type: rice fields, stagnant ditches and canals, holes in the ground, hoofprints, rock hollows, shallow or drying ponds. Some larvae have been collected in rivers, which is exceptional. *An. maculipalpis* is often found in the larval stage in association with *An. gambiae*, *An. squamosus*, and *An. mascarensis* ^4^. The pre-imaginal development period of this species is relatively long, at least one month. *An. maculipalpis* is generally considered zoophilic, exophagic, and exophilic ^4, 6^. In some localities, however, this species is reported to exhibit moderate anthropophily. This species is considered to have no medical importance, although a strain of the West Nile virus has been isolated in Madagascar, and experimental transmission of *Wuchereria bancrofti* has been achieved in the laboratory in Mauritius (Mascarene Archipelago, Indian Ocean) ^7^. Few studies have generated genetic sequences such as cytochrome oxidase subunit II (COII) or internal transcribed spacer 2 (ITS2) ^8, 9^. However, no population genetic work has been published, because of its no proven medical importance as a malaria vector.

The genome of the African malaria mosquito, *Anopheles maculipalpis*, was sequenced as part of the Anopheles Reference Genomes Project (PRJEB51690). Here we present a chromosomally complete genome sequence for *Anopheles maculipalpis*, based on a single wild-caught female.

## Genome sequence report

The genome was sequenced from a single female *Anopheles maculipalpis* collected in Lopé, Gabon (-0.142, 11.610) in April 2019. A total of 52-fold coverage in Pacific Biosciences single-molecule HiFi long reads (N50 10.236 kb) and 79-fold coverage in 10X Genomics read clouds were generated. Primary assembly contigs were scaffolded with chromosome conformation Hi-C data from an unrelated female individual.

**Figure 1.**
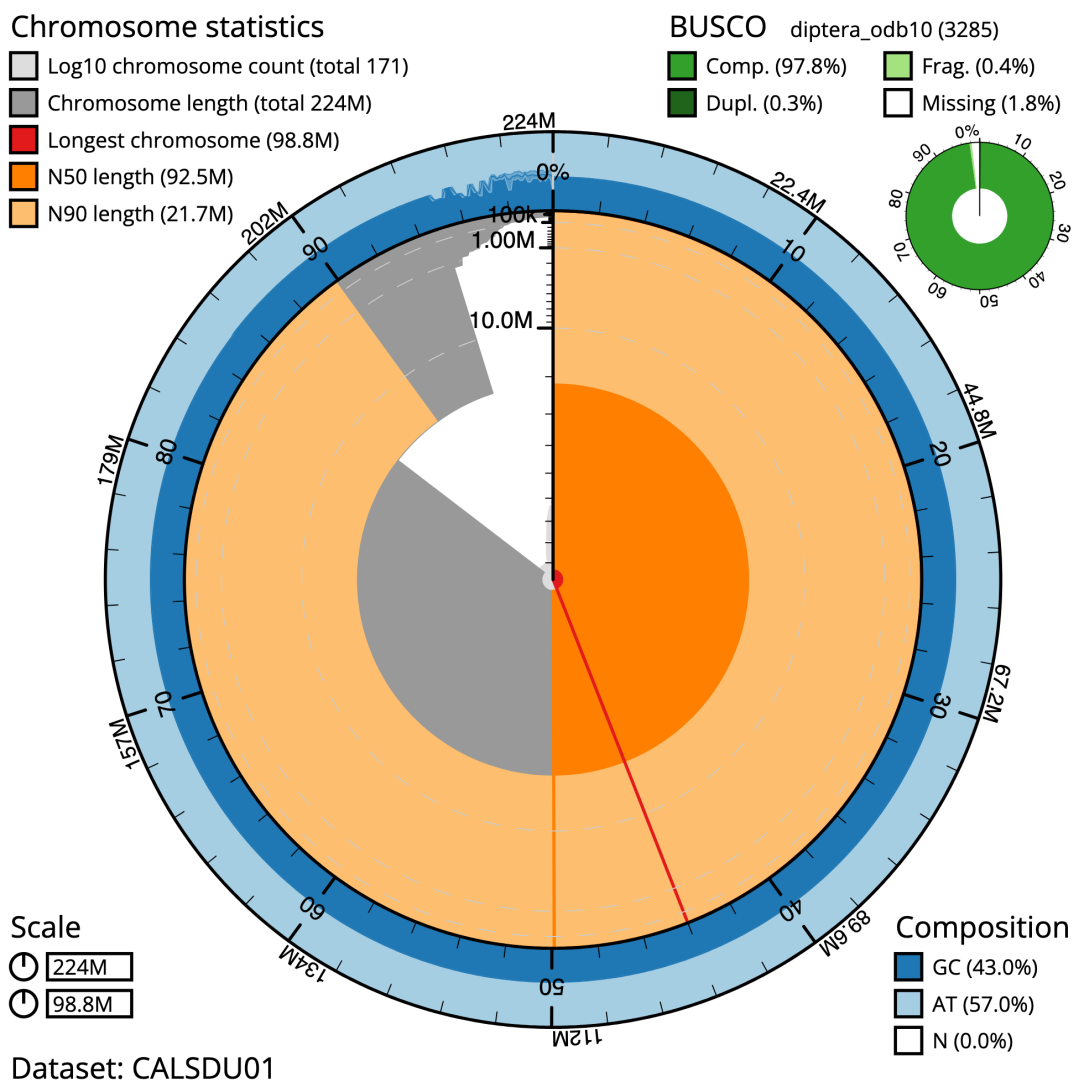
Snail plot summary of assembly statistics for *Anopheles maculipalpis* assembly. The main plot is divided into 1,000 size-ordered bins around the circumference with each bin representing 0.1% of the 224,074,805 bp assembly. The distribution of sequence lengths is shown in dark grey with the plot radius scaled to the longest sequence present in the assembly (98,751,411 bp, shown in red). Orange and pale-orange arcs show the N50 and N90 sequence lengths (92,548,589 and 21,651,475 bp), respectively. The pale grey spiral shows the cumulative sequence count on a log scale with white scale lines showing successive orders of magnitude. The blue and pale-blue area around the outside of the plot shows the distribution of GC, AT and N percentages in the same bins as the inner plot. A summary of complete, fragmented, duplicated and missing BUSCO genes in the diptera_odb10 set is shown in the top right. An interactive version of this figure is available at https://blobtoolkit.genomehubs.org/view/idAnoMacuDA_375_x/dataset/CALSDU01/snail.

**Figure 2.**
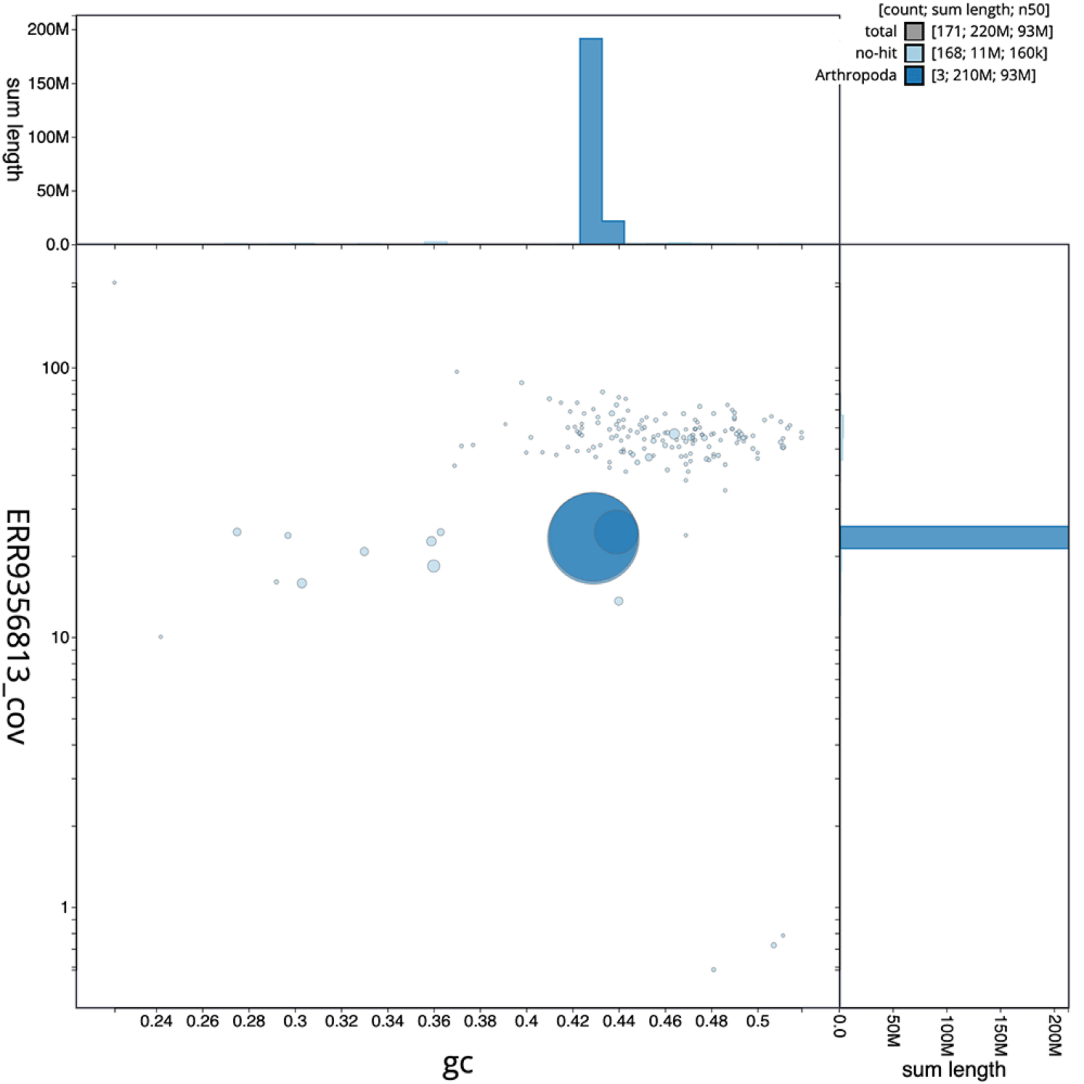
Blob plot of base coverage in a subset of idAnoMacuDA-375_x 10x linked reads against GC proportion for *An. maculipalpis* assembly idAnoMacuDA_375_x. Chromosomes are coloured by phylum. Circles are sized in proportion to chromosome length. Histograms show the distribution of chromosome length sum along each axis. An interactive version of this figure is available at https://blobtoolkit.genomehubs.org/view/idAnoMacuDA_375_x/dataset/CALSDU01/blob.

**Figure 3.**
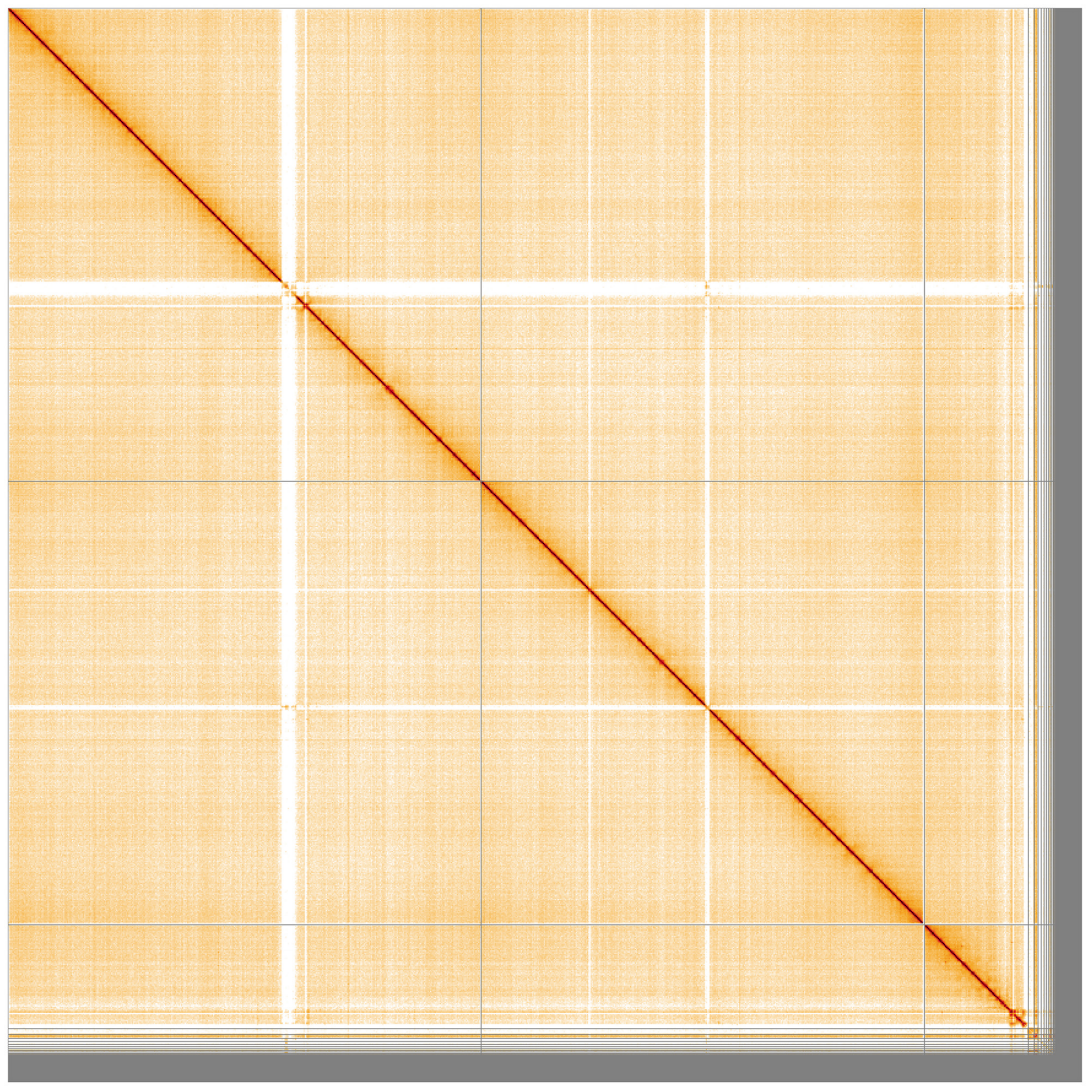
Genome assembly of *An. maculipalpis*, idAnoMacuDA_375_x: Hi-C contact map. Visualised in HiGlass. Chromosomes are ordered as 2RL, 3RL, X, then remaining scaffolds. The interactive Hi-C map can be viewed at https://genome-note-higlass.tol.sanger.ac.uk/l/?d=LR0ErKCBRiyLbpoq-qAnnw.

**Figure 4.**
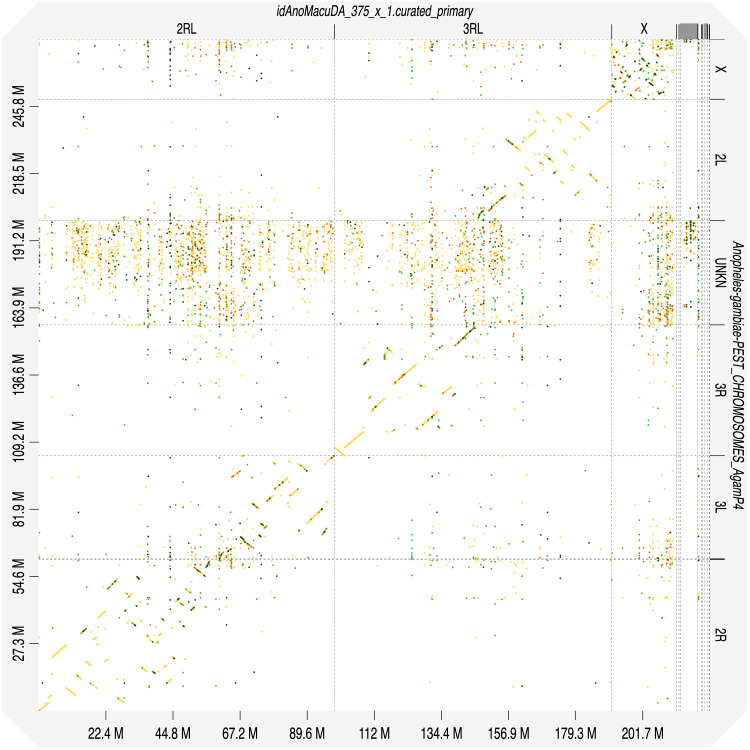
Alignment dotplot between genome assemblies of *An. maculipalpis* idAnoMacuDA_375_x and *An. gambiae*, AgamP4 (PEST). Chromosome arms correspondence (maculipalpus-gambiae): 2R-2R, 2L-3L, 3R-3R, 3L-2L in agreement with ^11^.

The final assembly has a total length of 224 Mb in 171 sequence scaffolds with a scaffold N50 of 92.549 Mb ( Table 1). The snail plot in Figure 1 provides a summary of the assembly statistics, while the distribution of assembly scaffolds on GC proportion and coverage is shown in Figure 2. 98.19% of the assembly sequence was assigned to three chromosomal-level scaffolds, representing two autosomes and the X sex chromosome ( Figure 3; Table 2). Chromosomes were numbered and oriented using synteny to the AgamP3 assembly ^10^ (accession GCF_000005575.2) ( Figure 4) based on cytogenetics data ^11^ ( Figure 4). The assembly has a BUSCO 5.3.2 ^12^ completeness of 97.8% using the diptera_odb10 reference set. While not fully phased, the assembly deposited is of one haplotype and also includes the circular mitochondrial genome. Contigs corresponding to the second haplotype have also been deposited.

**Table 1.** Genome data for *An. maculipalpis*, idAnoMacuDA_375_x.

*Project accession data*	
Assembly identifier	idAnoMacuDA_375_x
Species	*Anopheles maculipalpis*
Specimen	idAnoMacuDA-375_x
NCBI taxonomy ID	1496333
BioProject	PRJEB53251
BioSample ID	ERS10527369
Isolate information	female, whole organism
*Raw data accessions*	
PacificBiosciences SEQUEL II	ERR9439503
10X Genomics Illumina	ERR9356813, ERR9356814, ERR9356815, ERR9356816
Hi-C Illumina	ERR9356811, ERR9356812
PolyA RNA-Seq Illumina	ERR9356817
*Genome assembly*	
Assembly accession	GCA_943734695
Accession of alternate haplotype	GCA_943734695
Span (Mb)	224.075
Number of contigs	182
Contig N50 length (Mb)	22.618
Number of scaffolds	171
Scaffold N50 length (Mb)	92.549
Longest scaffold (Mb)	98.751
BUSCO * genome score	C:97.8%[S:97.5%,D:0.3%], F:0.4%,M:1,8%,n:3285

* BUSCO scores based on the diptera_odb10 BUSCO set using BUSCO 5.3.2. C=complete [S=single copy, D=duplicated], F=fragmented, M=missing, n=number of orthologues in comparison. A full set of BUSCO scores is available at https://blobtoolkit.genomehubs.org/view/idAnoMacuDA_375_x/dataset/CALSDU01/busco.

**Table 2.** Chromosomal pseudomolecules in the genome assembly of *An. maculipalpis*, idAnoMacuDA_375_x.

INSDC accession	Chromosome	Size (Mb)	Count	Gaps
OX030895.1	2RL	98.751	1	3
OX030896.1	3RL	92.549	1	3
OX030897.1	X	21.651	1	1
OX030898.1	MT	0.015	1	0
	X Unlocalised	7.073	155	3
	Unplaced	4.035	12	1

**Figure 5.**
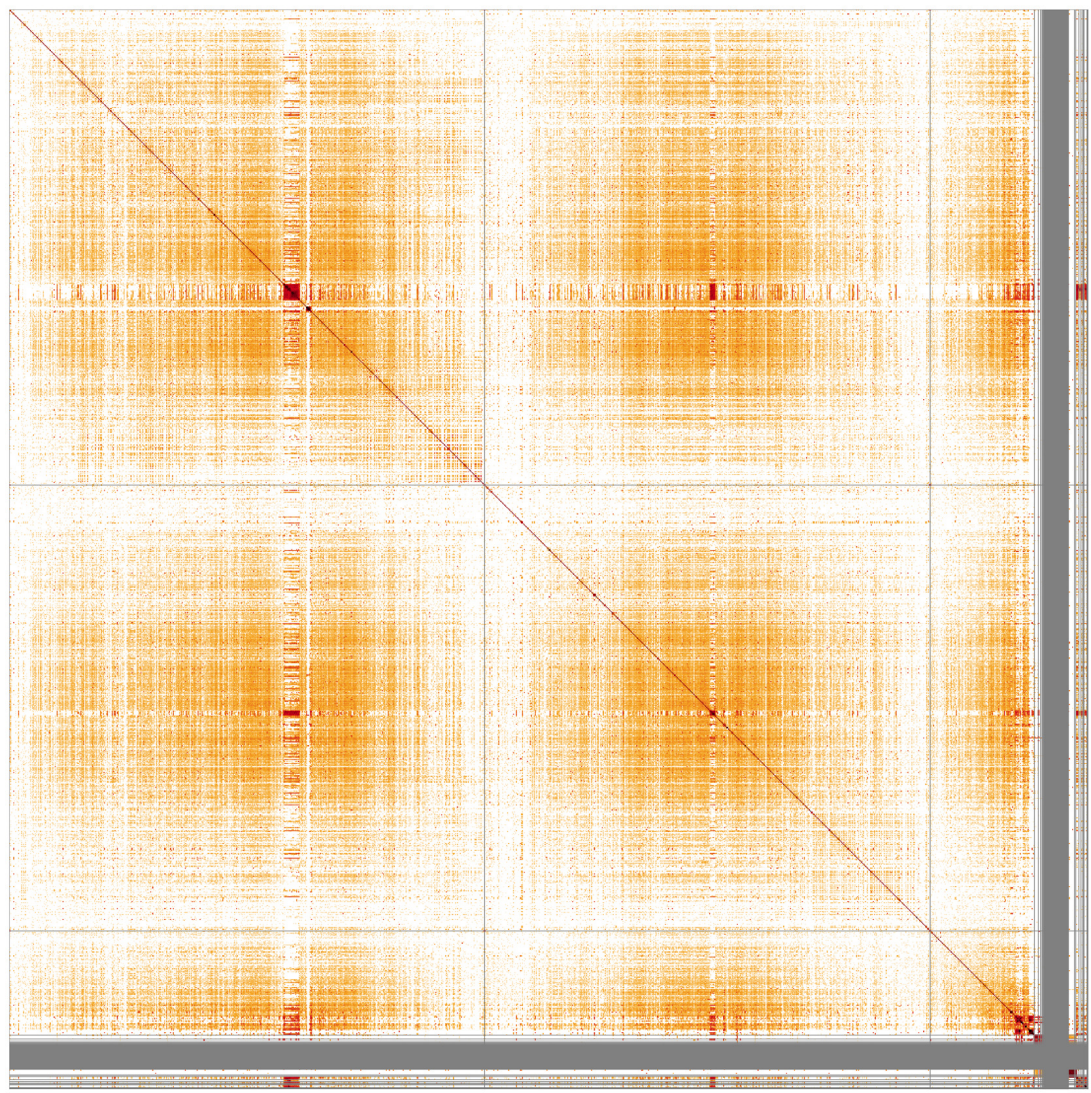
Sequence similarity heatmap for genome assembly of *An. maculipalpis*, idAnoMacuDA_375_x. Produced with StainedGlass, visualised in HiGlass. CChromosomes are ordered as 2RL, 3RL, X, then remaining scaffolds. Darker colours represent higher sequence similarity, notably at putative centromeres and terminal part of X.

Chromosome arms, candidate centromere and the rDNA regions were delineated based on the presence of characteristic tandem repeat arrays ( Figure 5; Table 3). Putative centromeres of autosomes comprised long stretches of irregular tandem repeats with varying unit lengths and arrangements, but with significant sequence homology between chromosomes 2 and 3. These locations were in agreement with the Hi-C contact map ( Figure 3) and synteny to *An. gambiae* ( Figure 4). Terminal part of chromosome X, starting at 17,790,992, also featured variable tandem repeats, which could be grouped into at least six different types based on sequence similarity. None of those could be reliably identified as centromeric. Arrays of rRNA genes were located in X-linked unlocalised scaffolds.

**Table 3.** Chromosome arms in the genome assembly of *An. maculipalpis*, idAnoMacuDA_375_x.

Chromosome	Start	End	Chromosome arm
2RL	1	57,070,844	2R
2RL	60,243,198	98,751,411	2L
3RL	1	46,889,622	3R
3RL	47,935,636	92,548,589	3L
X	1	21,651,475	X

Gene annotation was performed with NCBI Eukaryotic Genome Annotation Pipeline and is available in the RefSeq ^13^ under the accession GCF_943734695.1. A total of 12,929 genes were predicted, including 11,533 protein-coding genes and 1,092 non-coding RNAs. The genome assembly and gene annotations are hosted on VectorBase, www.vectorbase.org ^14^ under the identifier AmacGA1.

## Methods

### Sample acquisition and nucleic acid extraction

*Anopheles maculipalpis* individuals were caught using a human landing catch. A single female idAnoMacuDA-375_x was used for Pacific BioSciences and 10x genomics, an unrelated female idAnoMacuDA-405_x was used for Arima Hi-C.

For high molecular weight (HMW) DNA extraction one whole insect (idAnoMacuDA-375_x) was disrupted by manual grinding with a blue plastic pestle in Qiagen MagAttract lysis buffer and then extracted using the Qiagen MagAttract HMW DNA extraction kit with two minor modifications ^15^. The quality of the DNA was evaluated using an Agilent FemtoPulse to ensure that most DNA molecules were larger than 30 kb, and preferably > 100 kb. In general, single mosquito extractions ranged in total estimated DNA yield from 200 ng to 800 ng, with an average yield of 500 ng. Low molecular weight DNA was removed using an 0.8X AMpure XP purification. A small aliquot (less than ~5% of the total volume) of HMW DNA was set aside for 10X Linked Read sequencing and the rest of the DNA was sheared to an average fragment size of 12–20 Kb using a Diagenode Megaruptor 3 at speeds ranging from 27 to 30.Sheared DNA was purified using AMPure PB beads with a 1.8X ratio of beads to sample to remove the shorter fragments and concentrate the DNA sample. The concentration and quality of the sheared and purified DNA was assessed using a Nanodrop spectrophotometer and Qubit Fluorometer with the Qubit dsDNA High Sensitivity Assay kit. Fragment size distribution was evaluated by running the sheared and cleaned sample on the FemtoPulse system once more. The median DNA fragment size for *Anopheles* mosquitoes was 15 kb and the median yield of sheared DNA was 200 ng, with samples typically losing about 50% of the original estimated DNA quantity through the process of shearing and purification.

For Hi-C data generation, a separate unrelated mosquito specimen (idAnoMacuDA-405_x) was used as input material for the Arima V2 Kit according to the manufacturer’s instructions for animal tissue. This approach of using a sibling was taken in order to enable all material from a single specimen to contribute to the PacBio data generation given we were not always able to meet the minimum suggested guidance of starting with > 300 ng of HMW DNA from a specimen. Samples proceeded to the Illumina library prep stage even if they were suboptimal (too little tissue) going into the Arima reaction.

To assist with annotation, which will be made available through VectorBase ^14^ in due course, RNA was extracted from a separate whole unrelated female mosquito specimen (idAnoMacuDA-401_x) using TRIzol, according to the manufacturer’s instructions. RNA was then eluted in 50 μl RNAse-free water, and its concentration was assessed using a Nanodrop spectrophotometer and Qubit Fluorometer using the Qubit RNA Broad-Range (BR) Assay kit. Analysis of the integrity of the RNA was done using Agilent RNA 6000 Pico Kit and Eukaryotic Total RNA assay. Samples were not always ideally preserved for RNA, so qualities varied but all were sequenced anyway.

### Sequencing

We prepared libraries as per the PacBio procedure and checklist for SMRTbell Libraries using Express TPK 2.0 with low DNA input. Every library was barcoded to support multiplexing. Final library yields ranged from 20 ng to 100 ng, representing only about 25% of the input sheared DNA. Libraries from two specimens were typically multiplexed on a single 8M SMRT Cell. Sequencing complexes were made using Sequencing Primer v4 and DNA Polymerase v2.0. Sequencing was carried out on the Sequel II system with 24-hour run time and 2-hour pre-extension. A 10X Genomics Chromium read cloud sequencing library was also constructed according to the manufacturer’s instructions (this product is no longer available). Only 0.5 ng of DNA was used and only 25–50% of the gel emulsion was put forward for library prep due to the small genome size. For Hi-C data generation, following the Arima HiC V2 reaction, samples were processed through Library Preparation using a NEB Next Ultra II DNA Library Prep Kit and sequenced aiming for 100x depth. RNA libraries were created using the directional NEB Ultra II stranded kit. Sequencing was performed by the Scientific Operations core at the Wellcome Sanger Institute on Pacific Biosciences SEQUEL II (HiFi), Illumina NovaSeq 6000 (10X and Hi-C), or Illumina HiSeq 4000 (RNAseq).

### Genome assembly

Assembly was carried out with Hifiasm ^16^; haplotypic duplications were identified and removed with purge_dups ^17^. One round of polishing was performed by aligning 10X Genomics read data to the assembly with Long Ranger ALIGN, calling variants with FreeBayes ^18^. The assembly was then scaffolded with Hi-C data ^19^ using SALSA2 ^20^. The assembly was checked for contamination as described previously ^21^. Manual curation was performed using gEVAL ^22^, HiGlass ^23^ and Pretext ^24^. The mitochondrial genome was assembled using MitoHiFi ^25^, which performs annotation using MitoFinder ^26^. The genome was analysed and BUSCO scores were generated within the BlobToolKit environment ^27^. Synteny analysis was performed with D-GENIES ^28^. Repetitive sequences were visualised with StainedGlass ^29^ and tandem repeats were annotated with ULTRA ^30^. Table 4 contains a list of all software tool versions used, where appropriate.

**Table 4.** Software tools used.

Software tool	Version	Source
hifiasm	0.14	16
purge_dups	1.2.3	17
SALSA2	2.2-4c80ac1	20
longranger align	2.2.2	31
freebayes	1.3.1	18
MitoHiFi	2	25
gEVAL	N/A	22
HiGlass	1.11.6	23
PretextView	0.1.x	24
BlobToolKit	3.4.0	27
BUSCO	5.3.2	12
D-GENIES	1.4	28
StainedGlass	0.5	29
ULTRA	1.0.0 beta	30

## Ethics/compliance issues

The genetic resources accessed and utilised under this project were done so in accordance with the UK ABS legislation (Nagoya Protocol (Compliance) (Amendment) (EU Exit) Regulations 2018 (SI 2018/1393)) and the national ABS legislation within the country of origin, where applicable.

## Data Availability

European Nucleotide Archive: *Anopheles maculipalpis* genome assembly, idAnoMacuDA_375_x. Accession number PRJEB53251; https://identifiers.org/bioproject/PRJEB53251. The genome sequence is released openly for reuse. The *Anopheles maculipalpis* genome sequencing initiative is part of the Anopheles Reference Genomes project PRJEB51690. All raw sequence data and the assembly have been deposited in INSDC databases. Raw data and assembly accession identifiers are reported in Table 1.

## Author information

Members of Wellcome Sanger Institute Scientific Operations: Sequencing Operations are listed here: https://doi.org/10.5281/zenodo.12165051.
