# Supplementary material for: A chromosomal reference genome sequence for the malaria mosquito, Anopheles maculipalpis, Giles, 1902
Source: Wellcome Open Res. 2024 Sep 26;9:553. [Version 1] doi: 10.12688/wellcomeopenres.22988.1 (PMC11809154; doi:10.12688/wellcomeopenres.22988.1)
Supplement: Peer review history

# Reviewer report: Reviewer response for version 1

**DOI:** 10.21956/wellcomeopenres.25313.r114741

**Published:** 2024-12-30

**Authors:** Din Syafruddin, Puji Budi Setia Asih Setia Asih

**Affiliations:** 1 National Research and Innovation Agency (BRIN), Makassar, Indonesia; 2 Eijkman Research Center for Molecular Biology, National Research and Innovation Agency, Jakarta, Indonesia

**General comment:**

 The manuscript reports the complete genome sequence of *Anopheles maculipalpis,* one of the malaria vectors in Africa and in the islands of South-West Indian Ocean.  While the manuscript contains novel and Indexing data, the complete genome sequence of many species of Anopheles, such as *An. gambiae* and others have been published previously and are available in the database.  Therefore, rather than providing a genome sequence analysis,  data on RNAomics, particularly related to vector status, blood meal preference and other aspect related to disease transmission should be explored. 

**Specific comments:**

1. The title should not mention “chromosomal” only as the manuscript reports full chromosomal genome sequence of *Anopheles maculipalpis *as well as  the mitochondrial genome.
2. The phylogenetic tree of the *Anopheles maculipalpis* and other major malaria vector worldwide might be reconstructed, based on mitochondrial genome or internal transcribed spacer 2 (ITS2) gene.

Are sufficient details of methods and materials provided to allow replication by others?

Yes

Is the rationale for creating the dataset(s) clearly described?

Yes

Are the datasets clearly presented in a useable and accessible format?

Yes

Are the protocols appropriate and is the work technically sound?

Yes

Reviewer Expertise:

Molecular entomology and parasitology

We confirm that we have read this submission and believe that we have an appropriate level of expertise to confirm that it is of an acceptable scientific standard, however we have significant reservations, as outlined above.

# Reviewer report: Reviewer response for version 1

**DOI:** 10.21956/wellcomeopenres.25313.r115140

**Published:** 2024-12-23

**Authors:** Renee Ali

**Affiliations:** 1 Molecular Microbiology and Immunology, Johns Hopkins University Bloomberg School of Public Health, Baltimore, Maryland, USA

The rationale for this study is sound and would be useful as reference material for future comparisons with specimens from other endemic regions. The BUSCO score of 97.8%  indicates a high-quality gene content and the assembly is reasonably correct. The methods presented are clearly described and can be reproducible if implemented on other *Anopheles* mosquito species.

Are sufficient details of methods and materials provided to allow replication by others?

Yes

Is the rationale for creating the dataset(s) clearly described?

Yes

Are the datasets clearly presented in a useable and accessible format?

Yes

Are the protocols appropriate and is the work technically sound?

Yes

Reviewer Expertise:

entomology, mosquito population genomics, molecular biology

I confirm that I have read this submission and believe that I have an appropriate level of expertise to confirm that it is of an acceptable scientific standard.

# Reviewer report: Reviewer response for version 1

**DOI:** 10.21956/wellcomeopenres.25313.r114735

**Published:** 2024-12-17

**Authors:** Craig S Wilding

**Affiliations:** 1 University College Dublin School of Biology and Environmental Science (Ringgold ID: 123708), Dublin, Leinster, Ireland

Rahola et al. describe the sequencing of a single *Anopheles maculipalpis* individual, reporting a high quality (in terms of scaffold-length and BUSCO completeness) genome generated using a combination of PacBio and Hi-C scaffolding for genome sequencing, and Illumina for RNASeq. The methods utilized are reported clearly although there is one exception. The sample collection section of the method says "A single female idAnoMacuDA-375_x was used for Pacific BioSciences and 10x genomics, an unrelated female idAnoMacuDA-405_x was used for Arima Hi-C." Then the Hi-C methods section states "For Hi-C data generation, a separate unrelated mosquito specimen (idAnoMacuDA-405_x) was used as input material for the Arima V2 Kit according to the manufacturer’s instructions for animal tissue. This approach of using a sibling was taken in order to enable all material from a single specimen to contribute to the PacBio data generation given we were not always able to meet the minimum suggested guidance of starting with > 300 ng of HMW DNA from a specimen." My question is, how is this using a sibling if this was, as stated twice previously, an unrelated female?

 All data are deposited and freely available.

 The manuscript is clearly written and the background provides useful information on this species and the utility of the reported resource which should be of value for comparative genomics work and further research on this species.

Are sufficient details of methods and materials provided to allow replication by others?

Yes

Is the rationale for creating the dataset(s) clearly described?

Yes

Are the datasets clearly presented in a useable and accessible format?

Yes

Are the protocols appropriate and is the work technically sound?

Yes

Reviewer Expertise:

Evolutionary genetics

I confirm that I have read this submission and believe that I have an appropriate level of expertise to confirm that it is of an acceptable scientific standard.

# Reviewer report: Reviewer response for version 1

**DOI:** 10.21956/wellcomeopenres.25313.r114738

**Published:** 2024-12-16

**Authors:** Daibin Zhong

**Affiliations:** 1 University of California at Irvine, Irvine, USA

The study by Rahola et al. presents a high-quality chromosome-level genome assembly for Anopheles maculipalpis. Utilizing a combination of long-read and short-read sequencing technologies, including PacBio and Illumina, along with Hi-C scaffolding, the authors generated a robust genome assembly with a scaffold N50 of 92.549 Mb. A high BUSCO completeness score (97.8%) indicates a highly accurate and complete assembly. The paper is well-written and presents the results clearly and concisely. The experimental design and methodology are sound. This valuable genomic resource will significantly contribute to our understanding of An. maculipalpis biology and facilitate future research on malaria control strategies. I have no further comments.

Are sufficient details of methods and materials provided to allow replication by others?

Yes

Is the rationale for creating the dataset(s) clearly described?

Yes

Are the datasets clearly presented in a useable and accessible format?

Yes

Are the protocols appropriate and is the work technically sound?

Yes

Reviewer Expertise:

population genetics, molecular biology, genomics, and the interplay between parasites and their vectors.

I confirm that I have read this submission and believe that I have an appropriate level of expertise to confirm that it is of an acceptable scientific standard.

# Reviewer report: Reviewer response for version 1

**DOI:** 10.21956/wellcomeopenres.25313.r104027

**Published:** 2024-10-15

**Authors:** Komal Kumar Bollepogu Raja

**Affiliations:** 1 Department of Pathology & Immunology, Baylor College of Medicine, Houston, TX, USA

The manuscript by Rahola et al. provides a comprehensive overview of the genome sequencing and assembly of *Anopheles maculipalpis*. The authors have provided specific details about the ecological background of this species.  The methods section is thorough and provides clear descriptions of the techniques used and allow reproducibility. The genome assembly results including mitochondrial genome are well presented with specific statistics and references to figures and tables. The BUSCO completeness score of 97.8% indicates a very high-quality assembly. The authors have deposited their data and made it available to public. Overall, the manuscript is well written with the genomic data useful for research on comparative genomics and broad mosquito research community.

Are sufficient details of methods and materials provided to allow replication by others?

Yes

Is the rationale for creating the dataset(s) clearly described?

Yes

Are the datasets clearly presented in a useable and accessible format?

Yes

Are the protocols appropriate and is the work technically sound?

Yes

Reviewer Expertise:

Genomics, Single cell omics, Bioinformatics and Neurobiology

I confirm that I have read this submission and believe that I have an appropriate level of expertise to confirm that it is of an acceptable scientific standard.
